# Compound Fracture of the Alveolus and Maxillary

**Published:** 1891-06

**Authors:** C. H. Wells

**Affiliations:** Huntingdon, Que.


ARTICLE IV.

### COMPOUND FRACTURE OF THE ALVEOLUS AND MAXILLARY.

BY C. H. WELLS, L. D. S., HUNTINGDON, QUE.

In attempting to bring forward this subject this evening, I feel very much like the man that was shipping coal to Newcastle, but, from what I see from different parts of the country, I feel encouraged to think that I may say something that will be of use to some one, and, if so, I shall feel that my object is attained.

The first case to which I will invite your attention is that of Robert Lumsden, of Athelstan, P. Q., at that time eight years old.

This is a compound fracture of the lower jaw, from the effects of a blow of a club, which struck the jaw to the left of the centre, coming end-wise, the centre of the blow being just in front of the canine, and causing a complete fracture between the second temporary molar and the six-year molar, on the left side; also another at the canine, or between the canine and the first molar; and another, between the right canine and the lateral incisor; and the fourth, between the second molar on the right and the first molar. Then there was another fracture, extending from one of the fractures at the canines to the other, thus breaking the alveolus with one canine and four centrals completely out, which the mother brought me in her hand. After examining the case carefully, I consulted the physician who had brought them to my office, and he proposed that we disjoint the lower jaw entirely, and take it out, as he did not think it possible to save it. I thought that I could make an improvement on that. I gave the patient æther, got the fractures reduced as near as possible to their proper places, and had them held in place by assistants until I took a wax impression, when I made a capping plate to fit over all the lower teeth, which, you will remember, were all knocked out but the two permanent molars. I replaced them all but one lateral incisor, where the socket was gone altogether, and I left it out, which I much regret, as I saved all that I put in, and I believe might have saved that one also. I then riveted a V-shaped piece of plated steel to the capping plate, passed a bolt down through the head of the V, soldered it fast there, and carried it down through an iron plate well padded underneath the jaw. On this bolt I put a thumb-nut, that could be loosened or removed at will; this I allowed to remain three days, rinsing the mouth with antiseptic washes. I then removed it carefully, and rinsed the mouth well with antiseptics, and at the end of the first week the patient was living on fairly solid food. After this I only removed the plate once a week. At the end of the fifth week I removed the plate altogether, and discharged the patient. Strange to say, he has since erupted his bicuspids and canines, and they all came all right; also the second molars are in their proper place and position. The enlargement of the bone at the fractures is very slight, so slight that you would not notice there had been anything wrong.

The second case is that of Archie McEwan, of Ormstown, P. Q., who came to me on the 8th of April last; he had been kicked by a horse five weeks before, and had been attended by two eminent surgeons. The only teeth he could make meet together were the lower right canine on the outside of the first superior bicuspid, the molars and bicuspids of both sides of the mouth being outside those of the upper jaw, and on the left side; when the right were touching, they lacked more than one-fourth of an inch of coming up to the upper ones. I found the union so strong that I did not dare to break it again for fear of not getting a union, and so decided to draw them into place with pressure. I first passed a strong rubber round the two bicuspids on the right and the canine, also another over the second bicuspid on the right and the first molar on the left. At the same time I passed a very strong one round the central and lateral on the left, and the two bicuspids, on the same side. The patient wore this arrangement from 9 a. m. until 6 p. m., when I had the spaces nearly closed on the right, and completely on the left. I then took an impression, and made a capping plate similar to Lumsden's, but cutting the plaster teeth short on the right, and padding heavily under the left side, thus giving it a constant twisting pressure. This the patient wore for five days, and then returned with the articulation much improved, and in the condition shown in the plaster cast, having taken it off twenty-four hours before. You will observe that the molars and bicuspids of the right side were again springing out. I then passed another strong rubber band round the bicuspids on the right, and the first molar on the left, and in four hours had them again in their proper position. I then tied the two bicuspids firmly to the canine and lateral, and left this ligature there two weeks. The patient objecting to wear the plate and pads, on account of its inconvenience, I then fixed a bandage, cutting out a three-inch piece from the centre, and sewing to either end of this two six-inch pieces of the strongest elastic that I could get; then sewed the other pieces to this. My arrangement was complete. I then put the centre between the elastics under the chin, brought them up the side of the face over the top of the head, crossed them around to the back, passed them forward to the point of the chin, and sewed the ends together, and attached the bandages together at the side of the face, thus holding the whole bandage in position. The patient wore this about two weeks, which left his articulation the same as before the fracture. Everything was then taken off, and all remained in position, the patient being a little weak for a time.

The third case is that of David Armstrong, of Front River, N. Y. This is a case of a man thirty-eight years of age, and was caused by the kick of a horse. There were three fractures, although only two went completely through, the one on the left, as indicated by the pencil marks, united in fairly good position, but the one on the right was bad, as shown by the cast. The front end of the right side was thrown out and up so much, so that the only teeth that would meet at all were the lower canine and the first superior bicuspid; this is the position in which I first found it eleven weeks after the accident, and firmly united in this position. This case I treated with the capping plate, combined with the same apparatus as Lumsden's, moving the teeth inward on an inclined plane, and padding heavily on the left side, and keeping the screw well tightened. In ten days I had them in perfect position, and kept them there afterwards with dental floss ligatures, holding them this way for about three weeks, which proved a perfect success.

[Dr. Wells exhibited casts and photographs of the cases.]—*Dominion Dental Journal.*

